# Supplementary material for: The Impact of Cerebral Perfusion on Mesenchymal Stem Cells Distribution after Intra-Arterial Transplantation: A Quantitative MR Study
Source: Biomedicines. 2022 Feb 1;10(2):353. doi: 10.3390/biomedicines10020353 (PMC8962387; doi:10.3390/biomedicines10020353)
Supplement: Supplementary file 1 [file biomedicines-10-00353-s001.zip › biomedicines-1495938-supplementary.pdf]

Supplementary Material

Safety of Transplantation

Intra-arterial transplantation is an affective delivery route for stem cells administration; however, it can cause additional thromboembolic strokes in case of incorrect transplantation parameters used. Guzman et al. [1] analyzed this problem in detail in an excellent review in «Stroke» journal and identified several factors crucial for the transplantation outcome: cell type and size, cell dose, infusion speed, treatment window, and the extent of preservation of the natural arterial blood flow in the feeding vessel. With the use of selected for the object and cell type parameters the IA delivery can be safe. This is also confirmed by the results of the first clinical trials dedicated to the safety of IA MSCs transplantation in stroke patients [reviewed in [2]]. The infusion parameters used in this study were selected with regard to the literature data and based on our previous works [3,4], where we investigated and discussed in detail the safety of transplantation. In the current study, despite using the selected close-to-optimum parameters, we controlled the safety of transplantation for each rat by performing the diffusion weighted MR imaging (DWI) with apparent diffusion coefficient maps (ADC) calculation before and after IA administration of MSCs. This pulse sequence allows to visualize zones with the cytotoxic edema (regions with restricted diffusion of free water molecules) and is widely used in clinical practice and basic research for early diagnostics of cerebral ischemia [5–7]. The voxel size of DWI pulse sequence used in our study was 0.33 × 0.33 × 0.5 mm, which means that detection threshold for visualization of cerebral infarction was approximately 55 nanoliters. Additionally, we performed histological analysis of brain sections of rats after IA injection of MSCs to evaluate the possibility of neuronal death in the perfused regions and zones of cells' accumulation. To do this we performed TUNEL analysis for detection of apoptotic cells. Our results verified the MRI data and confirm that there were no apoptotic brain cells in the zones of MSCs accumulation after transplantation (except for the infarction area). The obtained results can be found in the Figure S1.

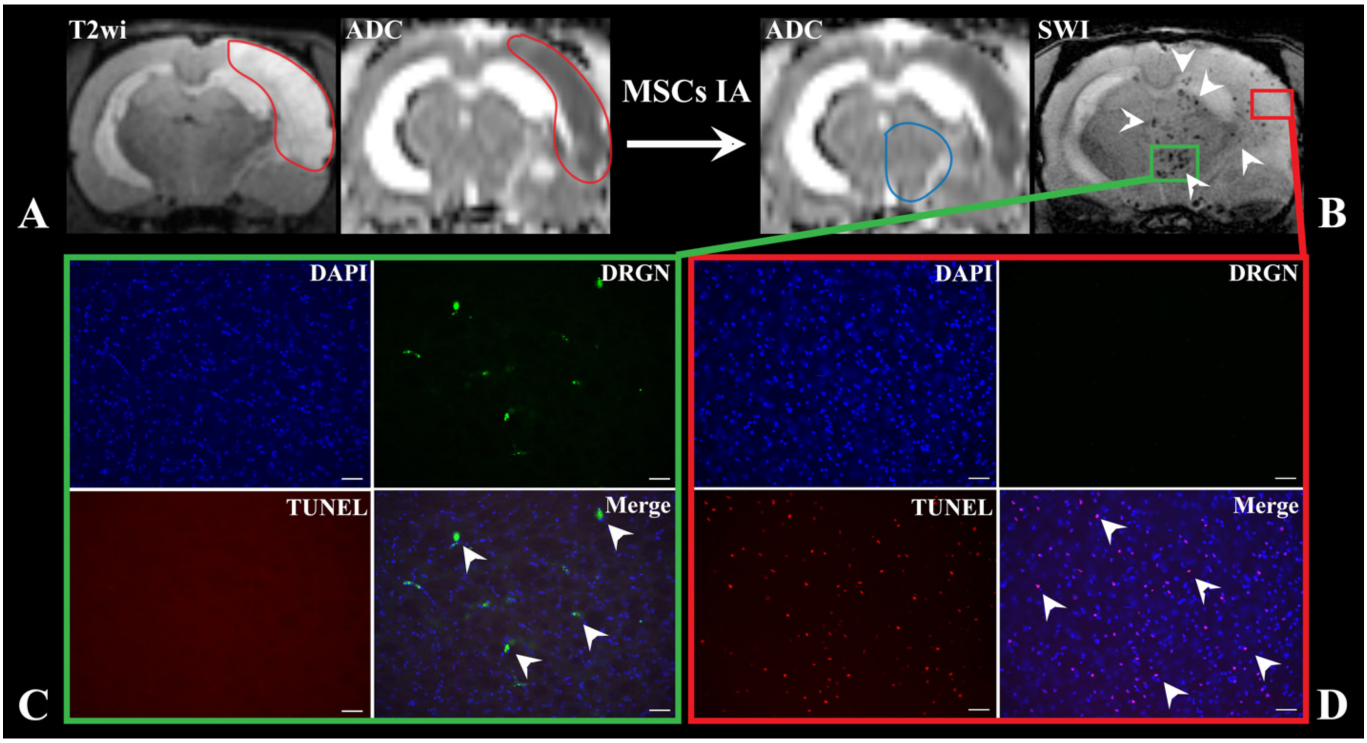

Figure S1. The safety of MSCs IA transplantation. The comparison of MRI and histological images of the brain of rat with experimental stroke after IA administration of labeled MSCs confirmed the absence of new infarction lesions formation. (**A**) T2 weighted images and Apparent Diffusion Coefficient (ADC) map before cells transplantation. The red lines indicate the ischemic lesion after MCAO stroke modeling, which is hyperintense on T2wi with restricted diffusion on ADC. (**B**) Apparent Diffusion Coefficient (ADC) map and SWI after cells MSCs IA transplantation. White arrows indicate labeled cells (hypointense dots on SWI). In the places of MSCs accumulation no additional lesions with restricted diffusion were detected (blue line area on ADC). (**C**) High-magnification fluorescent images of the rat brain

from the zone with labeled cells accumulation outside the ischemic lesion (marked by the green rectangle in B). Transplanted labeled MSCs marked with white arrows, SPIO microparticles with fluorescent marker Dragon Green - green, nuclei stained with DAPI - blue. TUNEL+ signal (red) of apoptotic cells is negative. Scale bars: 50 µm. (D) High-magnification fluorescent images of the rat brain in the ischemic lesion (marked by the red rectangle in B). TUNEL+ signal (red) of apoptotic cells marked with white arrows, nuclei are also stained with DAPI (blue). Scale bars: 50 µm.

References

1. Guzman, R.; Janowski, M.; Walczak, P. Intra-arterial delivery of cell therapies for stroke. *Stroke* **2018**, *49*, 1075–1082, doi:10.1161/STROKEAHA.117.018288.
2. Rascón-Ramírez, F.J.; Esteban-García, N.; Barcia, J.A.; Trondin, A.; Nombela, C.; Sánchez-Sánchez-Rojas, L. Are We Ready for Cell Therapy to Treat Stroke? *Front. Cell Dev. Biol.* 2021, *9*.
3. Namestnikova, D.; Gubskiy, I.; Gabashvili, A.; Sukhinich, K.; Melnikov, P.; Vishnevskiy, D.; Soloveva, A.; Vitushev, E.; Chekhonin, V.; Gubsky, L.; et al. MRI evaluation of frequent complications after intra-arterial transplantation of mesenchymal stem cells in rats. *J. Phys. Conf. Ser.* **2017**, *886*, 012012, doi:10.1088/1742-6596/886/1/012012.
4. Namestnikova, D.D.; Gubskiy, I.L.; Revkova, V.A.; Sukhinich, K.K.; Melnikov, P.A.; Gabashvili, A.N.; Cherkashova, E.A.; Vishnevskiy, D.A.; Kurilo, V. V.; Burunova, V. V.; et al. Intra-Arterial Stem Cell Transplantation in Experimental Stroke in Rats: Real-Time MR Visualization of Transplanted Cells Starting With Their First Pass Through the Brain With Regard to the Therapeutic Action. *Front. Neurosci.* **2021**, *15*, doi:10.3389/fnins.2021.641970.
5. González, R.G.; Schaefer, P.W.; Buonanno, F.S.; Schwamm, L.H.; Budzik, R.F.; Rordorf, G.; Wang, B.; Sorensen, A.G.; Koroshetz, W.J. Diffusion-weighted MR imaging: Diagnostic accuracy in patients imaged within 6 hours of stroke symptom onset. *Radiology* **1999**, *210*, doi:10.1148/radiology.210.1.r99ja02155.
6. Moseley, M.E.; Kucharczyk, J.; Mintorovitch, J.; Cohen, Y.; Kurhanewicz, J.; Derugin, N.; Asgari, H.; Norman, D. Diffusion-weighted MR imaging of acute stroke: correlation with T2-weighted and magnetic susceptibility-enhanced MR imaging in cats. *AJNR Am. J. Neuroradiol.* **1990**, *11*, 423.
7. Kleindorfer, D.O.; Towfighi, A.; Chaturvedi, S.; Cockroft, K.M.; Gutierrez, J.; Lombardi-Hill, D.; Kamel, H.; Kernan, W.N.; Kittner, S.J.; Leira, E.C.; et al. 2021 Guideline for the prevention of stroke in patients with stroke and transient ischemic attack; A guideline from the American Heart Association/American Stroke Association. *Stroke* 2021.
